# Hypogonadal hypertension in male Sprague-Dawley rats is renin-angiotensin system-dependent: role of endogenous androgens

**DOI:** 10.1186/s13293-020-00324-5

**Published:** 2020-08-26

**Authors:** Andrea E. Hanson, Mercedes Perusquia, John N. Stallone

**Affiliations:** 1 Department of Veterinary Physiology and Pharmacology, College of Veterinary Medicine and Biomedical Sciences, Texas A&M University, College Station, TX 77843-4466 USA; 2 Departamento de Biología Celular y Fisiología, Instituto de Investigaciones Biomédicas, Universidad Nacional Autónoma de México, 04510 México D.F, Mexico; 3 Michael E. DeBakey Institute For Comparative Cardiovascular Sciences, Women’s Health Division, College of Veterinary Medicine, Texas A&M University, College Station, TX 77843-4466 USA

**Keywords:** Anti-hypertensive, Angiotensin-converting enzyme, Blood pressure, Kidney, Non-genomic, Testosterone

## Abstract

**Background:**

Acutely, testosterone (TES) and other androgens are efficacious vasodilators, both in vitro and in vivo; however, their long-term effects on arterial blood pressure (BP) remain unclear. It was hypothesized that endogenous androgens exert long-term anti-hypertensive effects on systemic BP through a combination of genomic and nongenomic effects to enhance vasodilation of the systemic vasculature.

**Methods:**

The long-term effects of endogenous TES and exogenous TES replacement therapy (TRT) on BP were studied in intact (InT) and castrated (CsX) male Sprague-Dawley (SD) and testicular-feminized male (Tfm, androgen receptor defective) rats (12 weeks old). Systolic BP (tail-cuff plethysmography) was determined weekly for 15 weeks in InT-control and CsX rats. Some CsX-SD rats received androgen replacement therapy at 10-15 weeks with TES-enanthate (TRT; 1.75 mg/kg, 2x/week) or DHT-enanthate (DRT; 1.00 mg/kg. 2x/week) and a separate group of CsX-SD rats received losartan-potassium in drinking water (LST, 250 mg/L) for the entire 15 week period. Expression of renin, angiotensinogen (Agt), angiotensin converting enzyme (ACE), and angiotensin II type I receptor (AT_1_R) mRNA in kidney and aorta were determined by real-time PCR (rt-PCR) and plasma renin levels were determined by radioimmunoassay.

**Results:**

There was a progressive rise in BP over 10 weeks in CsX (109 ± 3.3 vs. 143 ± 3.5 mmHg), while BP remained stable in InT-control (109 ± 3.0 vs. 113 ± 0.3). BP gradually declined to normal in CsX-TRT rats (113 ± 1.3), while BP remained elevated in CsX (140 ± 1.2) and normal in InT-control (113 ± 0.3). LST prevented the development of hypertension in CsX at 10 weeks (100 ± 1.5 in CsX + LST vs. 143 ± 3.5 in CsX). During the next 5 weeks with TES-RT, BP declined in CsX-TRT (113 ± 1.3) and remained lower in CsX + LST (99 ± 0.4). DHT-RT reduced BP in CxS to a similar extent. In Tfm, CsX resulted in a similar rise in BP (109 ± 0.7 vs. 139 ± 0.4 mmHg), but TRT reduced BP more rapidly and to a greater extent (106 ± 2.8). rt-PCR of the kidney revealed that CsX increased expression of mRNA for renin (92%), ACE (58%), and AT_1_R (80%) compared to InT, while TES RT normalized expression of renin, AT1R, and ACE mRNA to levels of InT rats. Plasma renin levels exhibited changes similar to those observed for renin mRNA expression.

**Conclusions:**

This is the first study to examine the long-term effects of endogenous and exogenous androgens on BP in male SD and Tfm rats. These data reveal that endogenous androgens (TES) exert anti-hypertensive effects that appear to involve non-genomic and possibly genomic mechanism(s), resulting in reductions in RAS expression in the kidney and enhanced systemic vasodilation.

## Background

Cardiovascular disease (CVD) is a major cause of morbidity and mortality worldwide, and in the Western World, one-third of all deaths are attributed to CVD [1]. One of the most conspicuous characteristics of this serious healthcare problem is that most forms of CVD are higher in men than in age-matched premenopausal women, yet the reasons for these obvious sex differences remain poorly understood. The clinical studies and epidemiological observations that hypertension (HT) and coronary artery disease (CAD) occur more frequently in men than in premenopausal women [2–8] have led to the dogmatic view that testosterone (TES) and other androgens exert deleterious effects on the heart and vasculature, and exacerbate the development of CVD in men [9–11]. Indeed, the literature is replete with reports of the beneficial effects of estrogen on the female cardiovascular system [12–14]. In contrast, TES is usually considered to exacerbate CVD in males [9, 10, 15, 16]. In parallel, most animal studies in the past have provided support for the dogmatic view that TES exacerbates CVD [17–21]. For example, studies in both genetic (Dahl salt-sensitive and spontaneously hypertensive) and induced (DOCA-salt) rat models of HT reveal that castration ameliorates the development of HT in males [17, 22, 23].

However, more recent clinical and epidemiological studies on the role of TES in CVD are at best controversial, and in-depth reviews and analyses of the role of androgens in CVD reveal that there is little sound evidence that TES, or other androgens, shorten men’s lives [6, 8]. Interestingly, clinical studies over 70 years ago first documented the beneficial effects of TES on the cardiovascular system. Acute intramuscular injections of TES were used successfully in the treatment of angina pectoris in men with CAD [24–26], and for HT and peripheral vascular disease [26, 27] and were thought to involve vasodilation. Further, more recent clinical studies demonstrated that acute intracoronary injection or infusion of TES in men with CAD improves myocardial ischemia and increases coronary arterial dilation and blood flow [28–30].

In parallel, accumulating evidence from more recent animal studies employing isolated blood vessels in vitro, reveals that TES and other androgen metabolites exert beneficial effects by inducing acute vasorelaxation through rapid, nongenomic mechanisms in a variety of large arteries, as well as smaller resistance arteries, and at physiological as well as pharmacological concentrations (for reviews, see [31, 32]). There is also more limited evidence that TES and other androgens produce coronary or systemic vasodilation in humans [30] and in canine [33], porcine [34, 35], and rodent animal models [36]. Together, these in vitro and in vivo studies reveal that androgen-induced vasodilation is a structurally specific, nongenomic effect, with efficacies and potencies fundamentally different from those for the genomic effects of androgens on reproductive targets [31, 37–40].

Although the mechanisms underlying the acute vasodilatory effects of TES have been established in vitro and to a lesser extent in vivo, little is known about the long-term systemic effects of TES on blood pressure (BP). Thus in the present investigation, the long-term effects of endogenous and exogenous TES on arterial BP were determined in male Sprague-Dawley (SD) and testicular-feminized male [androgen receptor (AR)-deficient; Tfm] rats. The hypothesis tested was that endogenous androgens exert long-term anti-hypertensive effects on systemic BP through a combination of genomic and nongenomic effects to enhance vasodilation of the systemic vasculature. The results reveal that TES exerts clear anti-hypertensive effects on systemic BP that appear to involve genomic as well as nongenomic effects on the vasculature and kidney through inhibition of renin-angiotensin system expression.

## Methods

### Ethical approval

The animal use protocols for these studies were written in accordance with regulations of the Office of Laboratory Animal Welfare of the National Institutes of Health, and were reviewed and approved by the Texas A&M University Institutional Animal Care and Use Committee (IACUC).

### Experimental animals

Ten-week-old male Sprague-Dawley (SD) rats were purchased from Envigo (Houston, TX) and housed at the Laboratory Animal Resource and Research facilities at Texas A&M University in College Station, TX. The rats were maintained in well-ventilated rooms with controlled temperatures (21-26 °C), controlled relative humidity (~ 50%), and controlled photoperiod (12:12-h light: dark cycle). Animals were pair housed in standard plastic laboratory rat cages and fed a 16% protein rat chow that was soybean- and alfalfa-free (Teklad Global diet; Houston, TX). This diet is nominally free of dietary phytoestrogens, as ingested phytoestrogens have been reported to confound studies investigating sex differences in vascular function [41]. Water was provided ad libitum. The rats were randomly divided into one of three experimental groups: intact controls (InT-controls), castrated (CsX), or castrated with TES replacement therapy (CsX-TRT). Following arrival at Texas A&M University, all rats were acclimated to new housing for 4-5 days and then trained daily for handling and tail-cuff blood pressure recording for 7-10 days (see below), so that all rats were 12 weeks of age at the start of the experiments. One experimental group of SD rats studied (CsX-DRT, see below) were obtained from first-generation Envigo stock bred in-house, but were otherwise housed, handled, and treated identically as the rats obtained directly from Envigo. These rats were estimated to be 14 weeks of age at the start of the experiments.

Parallel studies using age-matched Tfm rats were used to separate the nongenomic and genomic effects of TES. This rat breed was originally derived from the King/Holtzman strain and affected males (termed Tfm rats) exhibit a recessive X-linked single gene mutation, leading to a defective cytosolic androgen receptor (AR) that eliminates the classical genomic effects of TES [42–44]. Affected males are genotypically male (XY), but phenotypically female with small abdominal testes and external female genitalia and produce elevated levels of TES. This phenotypic presentation is due to the loss of AR-mediated differentiation and the development of the male reproductive tract. These Tfm rats serve as a natural AR “knockout” model to investigate the genomic vs. non-genomic effects of endogenous and exogenous TES [36, 45, 46]. A breeding colony of these animals was established at Texas A&M University by breeding carrier female King/Holtzman stock to male SD rats and the resulting F1 carrier females were then bred to male SD rats; the resulting F2 Tfm males were used in the present experiments. Affected Tfm rats were randomly divided into one of three experimental groups: InT-control, CsX or CsX-TRT and experiments were initiated at 12 weeks age.

### Surgical procedures (bilateral orchiectomy)

Male SD rats in CsX and CsX-TRT groups underwent bilateral orchiectomy, using standard surgical procedures at 12 weeks age. Briefly, animals were pretreated with atropine sulfate (0.05 mg kg^−1^; *sc*) and anesthetized with isoflurane. Ophthalmic ointment was applied to the eyes of each animal as a preventative for corneal dryness. During the entire surgical procedure, animals were provided supplemental heat via a heating pad and continuously monitored for changes in respiratory rate, membrane color, and reflex response to ensure an adequate surgical plane of anesthesia. The surgical site was clipped, aseptically prepared, using alternating scrub cycles of iodine and 70% isopropyl alcohol, and the area was covered with a surgical drape. A single 1-2 cm incision was made in the scrotum and the muscular cremaster tunic which encloses each testis was bluntly dissected. Additional 1 cm incisions were made through the left and right tunics to externalize each of the testes. The spermatic cords were ligated with 1-0 surgical silk suture, transected, and the cut ends were swabbed with betadine and allowed to retract into the tunic. The tunics were sutured closed with absorbable sutures (Ethicon 4-0 Vicryl®), and the scrotum was closed with 3-5 absorbable sutures (Ethicon 4-0 Vicryl®). Banamine (2.5 mg kg^−1^; *im*) was administered for analgesia and the animals were transferred to a recovery cage.

Tfm rats in the CsX and CsX-TRT groups also underwent bilateral orchidectomy at 12 weeks age. Castration of Tfm rats involves a laparotomy to remove the abdominal testes in these animals. A small incision (2 cm) was made through the abdominal wall and the testicular arteries were isolated, ligated, and transected and the testes were then removed. The body wall musculature was closed with 3-4 absorbable sutures (Ethicon 4-0 Vicryl®), and the skin was closed with 3-4 stainless steel wound clips.

### Systolic blood pressure

Systolic BP was recorded using a non-invasive tail-cuff plethysmography system (IITC Life Science; Woodland Hills, CA) connected to a Power Lab Data Acquisition System and analyzed with the Lab Chart Software (AD Instruments; Colorado Springs, CO). Prior to data collection, animals were acclimated to daily handling and the tail-cuff procedure for 7-10 days and trained to rest quietly in a darkened cylindrical restraint warmed (30 °C) to maximize the tail blood flow signal. Week 0 systolic BP and body weight (BW) were recorded as baseline (time 0) data for each animal prior to castration or experimental treatments at 12 weeks age, and then weekly for 15 weeks following castration surgery.

### Experimental design

Weekly measurements of systolic BP and BW of Int, CsX and CsX-TRT SD, and Tfm rats began prior to CsX surgery (= baseline, time 0) and then resumed 7 days following surgery, and continued for the entire 15-week experimental period. Following BP and BW measurements on week 10, CsX-TRT rats began receiving bi-weekly injections of TES-enanthate dissolved in 90% olive oil/10% DMSO vehicle (1.75 mg kg^−1^; *sc*). CsX animals not receiving androgen therapy received bi-weekly injections of the olive oil-DMSO vehicle at equivalent volumes to the TES-enanthate. Systemically, TES can be reduced to dihydrotestosterone (DHT) or aromatized to estradiol (E_2_). To confirm that the observed effects of TES on BP regulation did not result from conversion to E_2_, the TES metabolite, 5α-DHT, which cannot be aromatized to E_2_, was administered as the androgen therapy in a separate group of CsX male SD rats (CsX-DRT). Following BP and BW measurements on week 10, CsX-DRT rats began bi-weekly injections of DHT-enanthate dissolved in 90% olive oil/10% DMSO vehicle (1.00 mg/kg; *sc*).

In a separate group of SD rats, losartan (LST), an angiotensin II type 1 receptor (AT_1_R) antagonist, was used to investigate the role of the renin-angiotensin-aldosterone system (RAS) in androgen-dependent changes in BP. Male rats were randomly assigned to one of three groups: CsX, CsX-LST, or CsX-LST-TRT. Animals were acclimated to weekly systolic BP measurements, and CsX animals underwent bilateral orchiectomy surgery as described previously. CsX-LST and CsX-LST-TRT groups received ad libitum drinking water with LST-potassium (250 mg L^−1^). Weekly systolic BP and BW were recorded for CsX, CsX-LST, and CsX-LST-TRT rats for the entire 15-week experimental period. CsX-LST-TRT animals began bi-weekly subcutaneous injections of TES-enanthate (1.75 mg kg^−1^; *sc*, 2x/week) following BP and BW measurements on week 10. CsX and CsX-LST animals not receiving androgen therapy received equivalent volumes of the olive oil-DMSO vehicle-control (*sc*, 2x/week).

### Plasma hormone concentrations

Animals were humanely euthanized via rapid decapitation at weeks 10 or 15 for plasma and tissue collection. Rapid decapitation minimizes potential neural and hormonal artifacts that result during anesthesia and euthanasia [47]. Trunk blood was collected in chilled 13 × 100 mm borosilicate glass test tubes containing 100-200 units of heparin sodium and immediately centrifuged at 10,000 RPM for 5 min to separate cells from plasma. The resultant plasma was frozen and stored at −80 °C for later analysis. Selective radioimmunoassays were used to assess plasma concentrations of TES and total estrogens (MP Biomedicals, LLC; Santa Ana, CA). Plasma renin was analyzed by Core Lab Facilities at Wake Forest University (Winston-Salem, NC). Plasma renin concentrations are defined as the rate of Ang I generated by renin in the plasma sample. In the presence of excess exogenous angiotensinogen isolated from nephrectomized rat plasma, renin concentrations were determined by incubating samples at a pH 6.5 for 90 min and quantifying Ang I by radioimmunoassay (Cisbio Bioassays; Codolet, France).

### RAS mRNA expression in the male rat aorta and kidney

The kidneys and thoracic aorta were collected from Int-control, CsX, and CsX-TRT rats at 0, 10, and 15 weeks and analyzed for RAS component mRNA expression by real time-polymerase chain reaction (rt-PCR). Following euthanasia, the thoracic aorta was immediately isolated, snap frozen in liquid nitrogen, and stored at −80 °C. The left kidney was isolated, cleaned of connective tissue and fat, sectioned, snap frozen on dry ice, and stored at −80 °C. Portions of the aorta and kidney samples (0.3-0.5 g) were used to analyze angiotensinogen (Agt), angiotensin-converting enzyme (ACE), AT_1_R, and renin mRNA expression using previously established rt-PCR methods [48]. Briefly, total RNA was isolated using the RNeasy Plus Mini Kit (Qiagen), according to manufacturer’s instructions. Individual RNA samples were tested for potency and total purified RNA samples (900-1500 ng) were seeded into a reverse transcription reaction using the SuperScript II system (Invitrogen), according to the recommended protocol for the high-capacity cDNA reverse transcription kit (Invitrogen), by combining 2.0 μL random hexamer oligonucleotides, 0.8 μL of 100 mM dNTP, 2.0 μL RT buffer, 1.0 μL MultiScribe™ reverse transcriptase, and 4.2 μL purified RNA plus water. Samples were brought to 25 °C for 5 min, 42 °C for 50 min, 45 °C for 20 min, 50 °C for 15 min, and 70 °C for 5 min. Relative levels of candidate gene transcripts were analyzed using the Dynamo Flash SYBR Green qPCR (Thermo Scientific), according to the recommended protocol. Reactions were quantified on a CFX38 touch RT-PCR detection system (BioRad). The primers for Agt, ACE, AT_1_R, renin, and Elongation Factor-1 (EF-1; reference gene) are listed in Table 1.

**Table 1:** Gene primers for RT-PCR

Gene	Sequence
Angiotensinogen (Agt)	Sense: AGCACGGACAGCACCCTATT
	Antisense: AGAACTCATGGAGCCCAGTCA
Angiotensin-converting enzyme (ACE)	Sense: CTGCCTCCCAACGAGTTAGAA
	Antisense: CGGGACGTGGCCATTATATT
Angiotensin II type I receptor (AT_1_R)	Sense: TATCACAGTGTGCGCGTTTCA
	Antisense: TGGTAAGGCCCAGCCCTAT
Renin	Sense: GCTACATGGAGAATGGGACTGAA
	Antisense: ACCACATCTTGGCTGAGGAAAC
Reference gene: elongation factor-1 (Ef-1)	Sense: GCAAGCCCATGTGTGTTGAA
	Antisense: TGATGACACCCACAGCAACTG

### Chemical reagents and drugs

The following drugs and reagents were used: TES-enanthate (17β-hydroxy-4-androsten-3-one-enanthate) and 5α-DHT-enanthate (17β-hydroxy-5α-androstan-3-one-enanthate; Steraloids; Newport, RI); and losartan-potassium (Selleck Chemical, Houston, TX). All other chemicals were of reagent grade quality and were purchased from Sigma Chemical (St Louis, MO). TES-enanthate and 5α-DHT-enanthate for androgen therapy experiments were dissolved in 90% olive oil/10% DMSO at 5.0 mg/ml.

### Data analyses

All data are expressed as the means ± standard error of the mean (SEM) and *n* indicates the number of animals in each experiment. BP data groups were first subjected to a two-way analysis of variance (ANOVA) with repeated measures to detect significant differences in BP among treatment groups (Int, CsX, CsX-TRT, CsX-DRT, CsX-LST, CsX-LST-TRT, and CsX-TRT-Tfm) and their interactions over time (0-15 weeks). BP data groups at key time points (0, 5, 10, 15 weeks) and other data groups (e.g., plasma renin or mRNA data) were first subjected to a one-way ANOVA to detect significant differences among means of the experimental groups; differences among means were accepted as significant if *P* value < 0.05. If the main effects were identified by the ANOVA, then pair-wise Student’s *t* tests were performed on group means to detect significant pair-wise differences among the means of the various experimental groups. A Bonferroni correction was employed for type I errors associated with multiple comparisons and differences between the means. Differences between any two means were accepted as significant if *P* < 0.05.

## Results

### Effects of castration and androgen therapy on systolic blood pressure

**Fig. 1:**
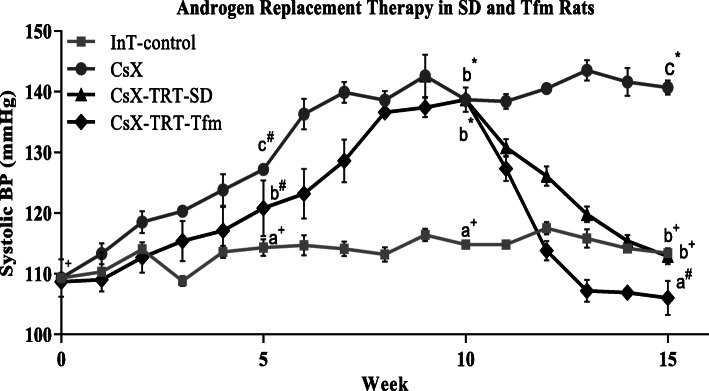
Weekly systolic blood pressure (BP) measurements of male Sprague-Dawley (SD) rats: intact (InT-control), bilaterally castrated (CsX), or CsX with TES therapy administered from weeks 10-15 (CsX-TRT; 1.75 mg kg^−1^; *sc*, 2x/week). Separate groups of testicular feminized male (Tfm) rats were CsX or CsX-TRT from weeks 10-15 (CsX-TRT-Tfm). Data points are means ± SEM (*n* = 5-10 rats per group). a, b, and c indicate that among the four experimental groups (Int-control, CsX, CsX-TRT-SD, and CsX-TRT-Tfm), mean values at each time point (5, 10, or 15 weeks) without common script are significantly different (0.0001 ≤ *P* ≤ 0.01). Plus sign, number sign, and asterisk for each experimental group (Int-control, CsX, CsX-TRT-SD, or CsX-TRT-Tfm), mean values over time (0, 5, 10, and 15 weeks) without common script are significantly different (0.0001 ≤ *P* ≤ 0.01)

Systolic BP of InT-control rats averaged 109 ± 3 mmHg at week 0. The repeated measures ANOVA revealed that BP differed significantly among the treatment groups and within each treatment group over time (*P* < 0.001). BP increased slightly the first 2 weeks of the experimental period and then maintained a stable plateau average of 114 ± 5 mmHg for the remainder of the 15-week experiment. In contrast, systolic BP of CsX rats exhibited a progressive and significant increase from week 0 (109 ± 3.0) to a maximal pressure at week 9 (143 ± 3.5), which then plateaued from weeks 10 to 15. Systolic BP of CsX at week 15 (140 ± 1.2) was significantly higher than InT-control (113 ± 0.3; *P* ≤ 0.0001). In a separate group of CsX, TRT administered from weeks 10 to 15 resulted in a progressive decline in systolic BP which completely normalized by week 15 to levels virtually identical to those of InT-control rats (CsX-TRT, 113 ± 1.3 vs. InT-control, 113 ± 0.3; *P* > 0.05). The effects of CsX on systolic BP were also determined in AR-deficient Tfm rats. Similar to the SD rats, castration of Tfm rats increased systolic BP, while TRT fully normalized BP. In contrast to the SD rats, Tfm rats exhibited a delay in the initial development of hypertension, but a more rapid onset of hypertension from weeks 5-10, compared to SD rats. Prior to CsX, systolic BP of CsX-Tfm rats averaged 109 ± 0.7 at week 0; following CsX, BP increased progressively and plateaued by week 10 (139 ± 0.4) and was fully normalized by TRT at week 15 (106 ± 2.8). TRT in Tfm rats resulted in a distinctly more rapid and greater overall decline in systolic BP compared to CsX-TRT SD rats. Weekly systolic BPs of InT-control, CsX, CsX-TRT, and CsX-TRT-Tfm rats are shown in Fig. 1.

### Mechanisms underlying anti-hypertensive effects of androgens on systolic BP

**Fig. 2:**
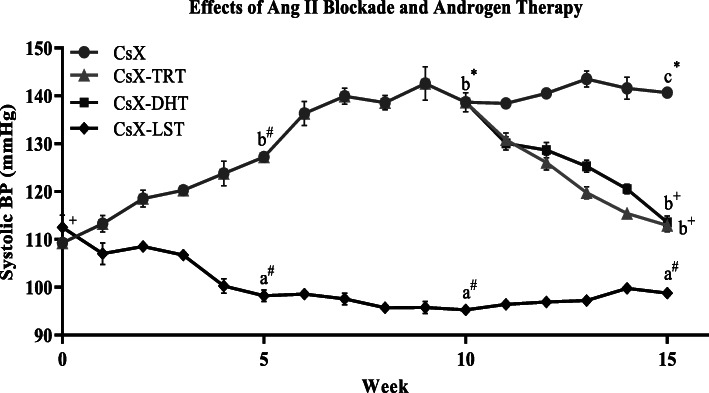
Weekly systolic blood pressure (BP) measurements of bilaterally castrated (CsX) male Sprague-Dawley (SD) rats: CsX with TES therapy administered from weeks 10-15 (CsX-TRT; 1.75 mg kg^−1^; *sc,* 2x/week), or CsX with DHT therapy administered from weeks 10-15 (CsX-DRT; 1.00 mg kg^−1^; *sc*, 2x/week). A separate group of CsX SD rats with TES therapy administered from weeks 10-15 (1.75 mg kg^−1^; *sc*, 2x/week) also received losartan-potassium (LST), an AT_1_R antagonist, in their drinking water (250 mg/L) for the entire 15-week experimental period (CsX-TES-LST). Data points are means ± SEM (*n* = 5-10 rats per group). a, b, and c indicate that among the four experimental groups (CsX, CsX-TRT, CsX-DHT, and CsX-LST), mean values at each time point (5, 10, or 15 weeks) without common script are significantly different (0.0001 ≤ *P* ≤ 0.02). Plus sign, number sign, and asterisk for each experimental group (CsX, CsX-TRT, CsX-DHT, or CsX-LST), mean values over time (5, 10, and 15 weeks) without common script are significantly different (0.0001 ≤ *P* ≤ 0.02)

The role of the RAS in the development of castration-induced hypertension is summarized in Fig. 2. Systolic BP declined significantly in CsX SD rats treated with the AT_1_R antagonist, losartan-potassium in the drinking water (CsX-LST; 250 mg L^−1^). Systolic BP declined until week 4 (CsX-LST, 100 ± 1.5 vs. CsX, 124 ± 2.6 mmHg; *P* ≤ 0.001), and remained significantly lower throughout the 15-week experimental period (CsX-LST, 99 ± 0.4 vs. CsX, 140 ± 1.2; *P* ≤ 0.0001). BP in an additional CsX-LST group also treated with TRT from weeks 10 to 15 (CsX-LST-TRT, 101 ± 0.8, data not shown) did not differ from CsX-LST (99 ± 0.4; *P* > 0.05). CsX rats receiving DHT androgen therapy (CsX-DRT) from weeks 10 to 15 exhibited declines in systolic BP that normalized identically to those of CsX-TRT rats (CsX-DRT, 114 ± 1.3 vs. CsX-TRT, 113 ± 1.3; *P* > 0.05).

### Effects of androgens on plasma renin concentrations

**Fig. 3:**
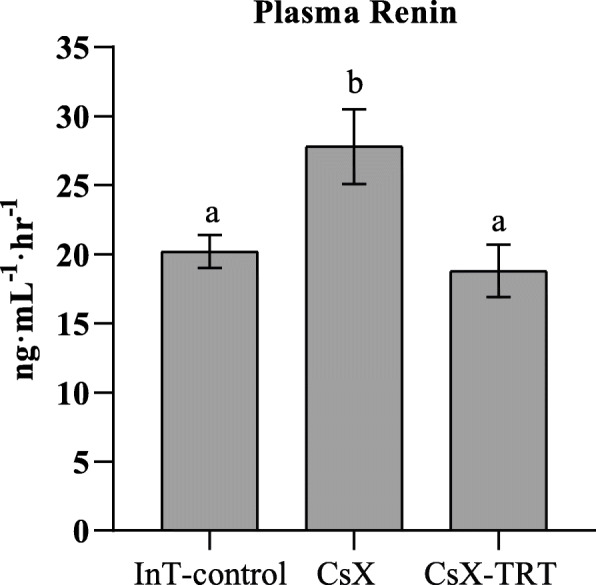
Plasma renin concentrations in male Sprague-Dawley (SD) rats: intact (InT-control), bilaterally castrated (CsX), or CsX with TES therapy (CsX-TRT; 1.75 mg kg^−1^; *sc*, 2x/week). Bars represent means ± SEM (*n* = 5-6 rats per group). a and b indicate the mean values for plasma renin without common superscript are significantly different (0.001 ≤ *P* ≤ 0.016)

Plasma renin concentrations are summarized in Fig. 3. CsX increased mean plasma renin concentration significantly (38%) compared to InT-control rats (*P* ≤ 0.02), while TRT reduced plasma renin concentration to levels similar to those of InT-control rats (*P* > 0.05).

### Effects of castration and androgens on expression of mRNA of RAS components

**Fig. 4:**
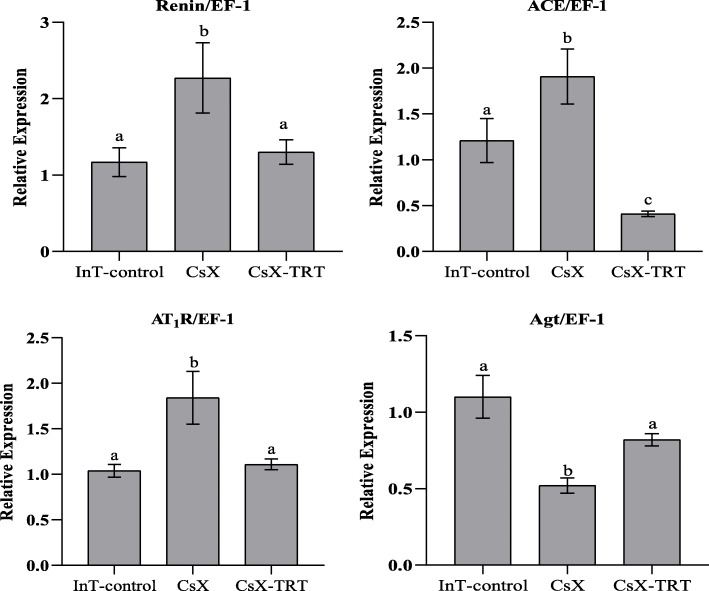
rt-PCR for mRNA expression of renin-angiotensin system (RAS) components in the kidneys of male Sprague-Dawley (SD) rats: intact (InT-control), bilaterally castrated (CsX), or CsX with TES therapy (CsX-TRT; 1.75 mg kg^−1^; *sc*, 2x/week). Values are expressed as the ratio of RAS component mRNA to elongation factor-1 (EF-1) mRNA levels obtained from the same tissues. Bars represent means ± SEM (*n* = 6-9 rats per group). a, b, and c indicate the mean values for mRNA expression of renin, angiotensinogen (Agt), angiotensin-converting enzyme (ACE), or angiotensin II receptor type I (AT_1_R) among the three experimental groups (InT-Control, CsX, and CsX-TRT) without common script are significantly different (0.0013 ≤ *P* ≤ 0.033)

mRNA expression of RAS components, as measured by rt-PCR, in the kidney and aorta of InT-control, CsX, and CsX-TRT male SD rats are shown in Fig. 4. In the kidney, CsX significantly increased expression of mRNA for renin (92%), AT_1_R (80%), and ACE (58%) compared to InT-control rats (0.0013 ≤ *P* ≤ 0.033), while TRT normalized expression of mRNA for renin, ACE, and AT_1_R to levels similar to those of InT-control rats (*P* > 0.05). In contrast, expression of mRNA for renal Agt was reduced by 55% in CsX rats compared to InT-control rats (*P* ≤ 0.0006), while TRT restored expression of Agt mRNA to 73% of InT-controls (*P* ≤ 0.01). In the thoracic aorta, neither CsX nor CsX-TRT had any statistically significant effects on expression of mRNA for renin, ACE, AT_1_R, or Agt (*P* > 0.05).

### Effects of androgens on body weight and seminal vesicle weight

Body weight (BW) and seminal vesicle (SV) weight for the seven experimental groups are summarized in Table 2. Week 0 BW was slightly lower in the InT-controls and higher in CsX-DRT compared to the other five experimental groups; however, BW increased similarly in all groups over the 15-week experimental period. BW was uniformly similar among most experimental groups at weeks 10 and 15, except for CsX-DRT and CsX-TES-Tfm, which were significantly higher (*P* ≤ 0.0001). Treatment with LST had no effect on BW (*P* > 0.05). SV weight varied significantly among the seven experimental groups (0.0001 ≤ *P* ≤ 0.0201). Castration reduced SV weight by more than 90% compared to InT-control rats (0.02 ± 0.001 g·100 g BW^−1^ vs. 0.29 ± 0.013), while SV weight in the CsX-TRT rats (0.33 ± 0.018) was fully restored and comparable to that of InT-control rats (*P* > 0.05). In contrast, DHT therapy only partially restored SV weight compared to CsX-TES rats (*P* < 0.05). Treatment with LST had no effects on SV weight in either CsX-LST or CsX-LST-TRT rats (*P* > 0.05).

**Table 2:** Body weight (BW) and seminal vesicle weight of InT-control, CsX, CsX-TRT, CsX-DRT, CsX-LST, CsX-LST-TRT, and CsX-TRT-Tfm male rats

Group	Week 0, BW (g)	Week 10, BW (g)	Week 15, BW (g)	Seminal vesicle (g·100 g of BW^**−1**^)
InT-control	339 ± 8^a+^	433 ± 12^a#^	439 ± 14^a#^	0.29 ± 0.013^a^
CsX	383 ± 22^b+^	457 ± 22^a#^	458 ± 27^a#^	0.02 ± 0.001^c^
CsX-TRT	374 ± 7^b+^	433 ± 7^a#^	446 ± 8^a#^	0.33 ± 0.018^a^
CsX-DRT	519 ± 13^c+^	594 ± 17^c#^	670 ± 26^c*^	0.10 ± 0.003^d^
CsX-LST	385 ± 4^b+^	456 ± 8^a#^	462 ± 11^a#^	0.03 ± 0.001^b^
CsX-LST-TRT	374 ± 3^b+^	432 ± 4^a#^	453 ± 5^a*^	0.34 ± 0.025^a^
CsX-TRT-Tfm	401 ± 23^b+^	487 ± 16^b#^	525 ± 20^b#^	

Male Sprague-Dawley (SD) rats: intact (InT), bilaterally castrated (CsX), and CsX with TES therapy (CsX-TRT; 1.75 mg kg^−1^; *sc*, 2x/week) or DHT therapy (CsX-DRT;1.00 mg kg^−1^; *sc*, 2x/week). In a separate experiment, CsX males were given losartan drinking water (250 mg L^−1^) without (CsX-LST) or with TES therapy (CsX-LST-TRT; 1.75 mg kg^−1^; *sc*, 2x/week). Testicular feminized male (Tfm) rats were castrated and given TES therapy (CsX-TRT-Tfm). Data are means ± SEM (*n* = 6-13 rats per group).

^a-c^Mean values within each column for BW (week 0, 10, and 15) and seminal vesicle weight without common superscript are significantly different (0.0001 ≤ *P* ≤ 0.02)

^+, #, *^Mean values for BW within each row for each of the experimental groups (InT-control, CsX, CsX-TRT, CsX-DRT, CsX-LST, CsX-LST-TRT, and CsX-TRT-Tfm) over time (week 0, 10, and 15) without common superscript are significantly different (0.0001 ≤ *P* ≤ 0.02)

### Effects of castration and androgen therapy on plasma sex hormone concentrations

The effects of castration and androgen therapy on plasma TES and estrogen concentrations are summarized in Table 3. As expected, castration drastically reduced the plasma TES concentration of CsX rats compared to InT-control male rats, while TRT restored plasma TES to levels similar to those of InT-control rats (*P* > 0.05). Plasma TES concentrations of CsX and CsX-LST rats were below detectable limits of the TES radioimmunoassay (< 0.01 ng mL^−1^). Plasma total estrogen concentrations were low among all experimental groups and were not significantly altered by CsX, TRT, or LST treatment (*P* > 0.05).

**Table 3:** Plasma total estrogen and testosterone (TES) of Int-control, CsX, CsX-TRT, CsX-LST, and CsX-LST-TRT male SD rats

Group	Total estrogen (pg mL^**−1**^)	Testosterone (TES) (ng mL^**−1**^)
InT-control	30.7 ± 0.6	0.72 ± 0.2
CsX	30.9 ± 1.2	---- a
CsX-TRT	31.3 ± 0.5	0.96 ± 0.1
CsX-LST	32.7 ± 0.2	---- a
CsX-LST-TRT	33.2 ± 0.6	1.14 ± 0.1

Male Sprague-Dawley (SD) rats: intact (InT-control), bilaterally castrated (CsX), and CsX with TES therapy (CsX-TRT; 1.75 mg kg^−1^; *sc*, 2x/week). In a separate experiment, CsX males were given drinking water with losartan (250 mg L^−1^) without TES therapy (CsX-LST) or with TES therapy (CsX-LST-TRT; 1.75 mg kg^−1^; *sc*, 2x/week). Data are means ± SEM (*n* = 6-13 rats per group). Neither total estrogen nor TES concentrations differed significantly among the five experimental groups (*P* > 0.05)

^a^Plasma TES levels in CsX and CsX-LST groups were below the detectable limits of the radioimmunoassay

## Discussion

In the present investigation, the long-term effects of endogenous and exogenous TES on systemic BP were studied in male SD and Tfm rats. The results reveal that endogenous as well as exogenous androgens (TES and 5α-DHT) exert novel long-term anti-hypertensive effects on systemic BP that appear to involve estrogen-independent, nongenomic, and possibly genomic mechanism(s), which reduce RAS expression in the kidney. Thus, the long-term anti-hypertensive effects of TES may involve enhanced fluid and electrolyte excretion as well as systemic vasodilation. Castration of InT-control male SD rats resulted in a dramatic reduction in plasma TES concentration and seminal vesicle mass (an important target tissue for TES), while TRT restored plasma TES concentration and seminal vesicle mass to nearly identical values as InT-control male SD rats. Additionally, there were no significant differences in plasma total estrogen concentrations among InT-control, CsX, and CsX-TRT male SD rats. Thus, TRT provided physiological replacement of circulating TES concentrations without any measurable changes in plasma estrogen levels. While the dose of TES therapy employed in the present study produced physiological concentrations and biological effects on reproductive target tissues (i.e., SV mass), the dose of DHT employed for androgen therapy only partially restored SV mass. Thus, the dose of DHT employed was either too low and/or systemic administration of DHT may not be as effective as the local conversion of TES to DHT that occurs within the SV, since DHT is a local tissue hormone and not a circulating systemic androgen. It should be noted, however, that the dose of DHT employed did exert virtually identical anti-hypertensive effects on systemic BP as TES over the 5 week treatment period; thus, the anti-hypertensive effects of TES observed in the present studies do not likely involve aromatization of TES to estrogen.

### Effects of castration and androgen therapy on systolic BP

In the present study, castration of male SD rats produced a progressive increase in systolic BP that attained a plateau after 10-12 weeks that continued through the entire 15 week experimental period. Androgen therapy of CsX rats with either TES or 5α-DHT at physiological levels beginning at 10 weeks post-CsX completely reversed the HT and restored systolic BP to levels comparable to those of normotensive InT-control male SD rats. These findings are similar to recent reports that castration produced similar levels of HT in both Wistar and Wistar-Kyoto male rats [49] and that in this model, acute *iv* infusions of TES and its metabolites produced reductions in the HT [50], although this latter study did not examine long-term effects of androgen therapy on the HT. The results of the present study are the first to demonstrate that both endogenous and exogenous TES exert long-term anti-hypertensive effects in male SD rats. In contrast, there is limited evidence that acute intra-arterial infusions of TES and other androgens produce coronary or systemic vasodilation in vivo in humans [30] and in canine [33], porcine [34, 35], and rodent [36] animal models. In parallel, accumulating evidence from a number of recent animal studies employing isolated blood vessels in vitro, reveals that TES and other androgen metabolites exert beneficial effects by inducing acute vasorelaxation through rapid, nongenomic estrogen-independent mechanisms in a variety of large arteries as well as smaller resistance arteries and at physiological as well as pharmacological concentrations (for reviews, see [31, 32]). Together, these in vitro and in vivo studies reveal that acute androgen-induced vasodilation is a structurally specific, nongenomic effect, with efficacies and potencies fundamentally different than those for the genomic effects of androgens on reproductive targets [31, 37–40]. An intriguing aspect of the structurally specific nongenomic effects of the androgens on vascular function is the effects of the endogenous TES metabolite, 5β-DHT, the stereoisomer of the highly potent androgenic tissue metabolite of TES, 5α-DHT, which is known to mediate the effects of TES in reproductive target tissues. In contrast, 5β-DHT is devoid of genomic androgenic activity at the androgen receptor, but is a highly potent and efficacious nongenomic vasodilator in vitro and in vivo [31, 36] and tissue levels of the enzyme that converts TES to 5β-DHT (5β-reductase) are reduced in patients with essential hypertension [51]. Thus, it is interesting to speculate that the TES metabolite of 5β-reductase, 5β-DHT, which is significantly more potent and efficacious than TES, but without any masculinizing side effects, may be an effective treatment for HT.

Although the mechanisms underlying the acute vasodilatory effects of TES have been established in vitro and to a lesser extent in vivo, little is known about the long-term systemic effects of TES on BP or its underlying mechanisms. To study these mechanism(s), the androgen receptor-deficient Tfm rat was used in the present study to identify possible non-genomic mechanisms contributing to the anti-hypertensive effects of TES. Interestingly, castration of Tfm rats induced long-term HT very similar to that observed in CsX SD male rats, and therapy with TES completely reversed the HT, indeed at a faster rate and to a slightly greater extent than in CsX male SD rats. These findings strongly suggest that the anti-hypertensive effects of endogenous and exogenous TES on systemic BP mainly involve nongenomic mechanisms. Nongenomic mechanisms may involve membrane receptors and include activation of kinase-signaling cascades or modulation of membrane ion channel function. In the present study, there was a progressive increase in systolic BP in both CsX male SD and CsX-Tfm rats; however, there was a delay in the onset of HT in the CsX-Tfm rats, followed by a more rapid increase in the HT which matched that of CsX-SD male rats by week 10, and a more rapid anti-hypertensive response to TES therapy. These differences suggest that interactions between the nongenomic and genomic effects of TES in male SD rats may be responsible for the observed differences between SD and Tfm rats in the development of HT and the anti-hypertensive responses to TES therapy.

### Mechanisms underlying anti-hypertensive effects of androgens on systolic BP

The long time-frame for the development of HT in castrated SD male and Tfm rats in the present study suggests that expansion of extracellular fluid volume resulting from the retention of fluid and electrolytes in the absence of TES may contribute to the HT. Thus, the role of RAS function in the development of HT was studied. Interestingly, long-term treatment of CsX male SD rats with LST, a competitive antagonist of the AT_1_ receptor, via their drinking water completely prevented the development of HT in these animals over the course of the 15-week experiment, strongly suggesting a role for the RAS in the development of HT in CsX-SD male rats. Consistent with this finding is the elevation in plasma renin concentration observed in these animals, which increased by 38% following CsX. Further support for a role of increased RAS function in the development of HT in these animals is provided by the observed increases in expression of mRNA for renin (92%), AT_1_R (80%), and ACE (58%) in the kidneys of CsX male SD rats. The observed increases in plasma renin concentration and in expression of renal mRNA for renin, ACE, and AT_1_R were dramatically reversed following TRT of CsX male SD rats in parallel with the complete reversal of systemic HT over the 5-week course of androgen therapy in these animals. These findings provide strong and consistent evidence that the development of long-term HT in CsX male SD rats in the absence of TES involves upregulation of RAS function in the kidneys of these animals, and that therapy with TES (or DHT) and the reversal of the HT involves inhibition of renal RAS expression by TES. Since there is now good evidence that TES produces systemic hypotension through nongenomically-mediated systemic vasodilation, it is likely that the observed development of HT in CsX male SD rats in the present study involves both the loss of TES-induced systemic vasodilation and expansion of extracellular fluid volume from the retention of fluid and electrolytes resulting from upregulation of renal RAS function in the absence of TES. Preliminary measurements of 24 h urine flow rates in male SD rats suggest that CsX reduces urine flow rate at experimental weeks 4 and 10, compared to Int-control rats, and that initiation of TRT in CsX-SD rats after week 10 measurements increases urine flow rates at weeks 12 and 15; findings consistent with the CsX-induced changes in systolic BP and RAS function (data not shown; ref. [52]). Studies by other investigators support the present hypothesis that the anti-hypertensive effects of TES involve its effects to suppress RAS function and thereby enhance renal fluid and Na^+^ excretion; thus, earlier studies by Kau et al. revealed that castration-induced loss of TES in male rats increased plasma aldosterone levels by nearly 40%, while TES produced dose-dependent reductions in basal and ACTH-stimulated aldosterone secretion by rat zona glomerulosa cells in primary culture [53]. Similarly, in more recent preliminary studies, expression of mRNA of key Na+ transporters (ENaC, NKCC, NCC, and NHE) in the renal cortex was 0.5-2.5 fold higher in CsX than in intact or CsX-TES male ANGII-hypertensive CD-1 mice [54]. Finally, the cytosolic AR is expressed in both the proximal tubule and cortical collecting duct of human kidneys, clearly implicating the kidney as a target for endogenous androgens [55].

Despite the aforementioned evidence that TES exerts anti-hypertensive effects on the kidney, there is also evidence to the contrary that androgens exert pro-hypertensive effects on the kidney; thus, in vivo renal microperfusion studies by Quan et al. established that 10-day treatment of SD rats with DHT enhanced volume reabsorption in the proximal tubule 38%, expression of the luminal Na^+^/H^+^ (NHE3) exchanger 55%, and mean arterial BP 18% [56]. These consistent renal effects of DHT were reduced by inhibition of converting enzyme (Enaliprilat) or blockade of the AT_1_R (LST), implicating the RAS in the pro-hypertensive effects of DHT. The physiological relevance of these findings is unclear, however, because DHT treatment reduced plasma ANG II levels by 30% and the dose of DHT employed (50 mg/day × 10 days) was dramatically higher than the physiological dose of DHT used in the present study (~ 1.0 mg/week × 5 weeks). Since human and rat kidney tissues both express enzymes involved in androgen biosynthesis and/or are capable of converting precursor compounds to TES and 5α-DHT, local production of androgens within the kidney may also play a role in BP regulation [57, 58].

The fact that HT develops following castration of the androgen receptor-deficient Tfm rats provides strong evidence that the anti-hypertensive effects of TES on the vasculature and kidney rely upon nongenomic mechanism(s) independent of the classic cytosolic AR that mediates the genomic effects of this hormone. In reproductive tissue cultures, AR-independent actions of TES are mediated by membrane receptors that can bind TES, such as the ZIP9 Zinc transporter [59] or other membrane-associated androgen receptor proteins [60]. These receptors appear to mediate the effects of TES via rapid activation of ERK1/2, Akt, and CREB signaling cascades. Similarly, there is evidence that a membrane-associated G protein-coupled TES receptor mediates AR-independent effects as well as modulates genomic effects of TES via ERK1/2/Akt pathways [59–61]. One of these receptors has been associated with beneficial effects of TES in the heart via the PI3/Akt pathway [62]. Since nongenomic mechanisms of TES action appear to involve membrane receptors and intracellular signal transduction cascades, and these signaling mechanisms may also modulate genomic expression, then it is still possible that a combination of genomic as well as nongenomic mechanisms may underlie the antihypertensive effects of endogenous TES in Tfm as well as SD male rats; however, resolution of this possibility must await further studies.

### Physiological relevance of the present findings

There is a firmly entrenched dogma surrounding CVD that estrogens are protective in females [12–14], whereas androgens are deleterious in males [9, 10, 15, 16]. These views appear to be based largely on earlier epidemiological data that have driven human clinical and experimental animal studies which support the dogmatic view that TES is deleterious to the heart and vasculature [9–11]; however, many of those past animals as well as human studies suffer from flaws or limitations in experimental design and/or the animal models employed [6, 8, 63]. More recently published clinical and experimental studies, as well as the findings of the present study, challenge the TES dogma. Indeed, there is increasing evidence that TES therapy in aging hypogonadal men significantly improves cardiovascular and metabolic functions, including a reduction in diastolic blood pressure [63–65]. Further, plasma TES levels are reduced in both hypertensive men and women [4, 66–68]. It is difficult to reconcile the enigma of beneficial effects of TES reported in more recent clinical studies with the many past experimental animal studies which support the dogma of deleterious effects of TES [17–21], unless more careful scrutiny is applied to these studies. Thus, widely used genetic and induced rat models of HT, such as the spontaneously hypertensive rat (SHR) and Dahl salt-sensitive rat [17, 23] and the DOCA-salt rat [22], respectively, have demonstrated repeatedly that TES does exacerbate HT in males, since castration of males reduces the development of HT. However, these models do so in an unrealistic experimental setting that does not mimic human hypertensive disease, since the effects of TES are determined on a background of established HT (genetic models) or simultaneously developing HT (induced models), rather than on a background of normotension, followed by progressive declines in TES levels and subsequent development of HT, as occurs in human males. Further, the short-term nature of these experiments (often 1-4 weeks) is problematic to the detection of longer term effects of TES on BP in otherwise normal male rats. These limitations may explain why findings from these established models of HT are so incongruous with recent clinical findings from aging human males in which hypogonadism is associated with cardiovascular and metabolic dysfunctions, including hypertension, and that TES therapy is beneficial in reversing these conditions.

### Clinical relevance of the present findings

The most recent human clinical trials overwhelmingly report that TRT does not increase cardiovascular risk or mortality in older hypogonadal men and that such therapy is associated with reductions in BP and improvement in metabolic syndrome (e.g., insulin resistance, serum lipids, diabetes, etc.), all of which are risk factors for CVD (for reviews see [63, 69, 70]). Indeed, of nine meta-analyses reported to date, all but one demonstrated that TRT is not harmful and is associated with significant health benefits [69, 70]. The findings of the present study are entirely consistent with these recent human clinical trials, and with much earlier studies in which acute intramuscular injections of TES were used successfully to treat angina pectoris in men with CAD [24–26], and for HT and peripheral vascular disease [26, 27], and were thought to involve vasodilation, and with more recent clinical studies in which intra-arterial infusions of TES produced coronary vasodilation [28–30]. Taken together, the past and present studies strongly suggest that TES and other androgens are, in reality, anti-hypertensive and thus protective against CVD.

## Conclusions

The findings of the present study provide important new and novel information on the long-term role of TES in the regulation of systemic BP, and reveal that endogenous androgens exert anti-hypertensive effects that appear to involve non-genomic and possibly genomic mechanism(s), resulting in reductions in RAS expression in the kidney and enhanced systemic vasodilation. These data further suggest that it is the age-dependent declines in TES levels and the effects of aging in men that exacerbate CVD, not the presence of TES. The effects of aging and hypogonadism on the development of HT in the model developed in the present study await further study, which may serve as a useful translational model to further elucidate the long-term role of TES in cardiovascular and metabolic health and disease.

## Perspectives and significance

The long-standing dogmatic view that TES is deleterious to the heart and vasculature [9–11] is challenged by the findings of the present study and by recent clinical findings from aging human males in which hypogonadism is associated with cardiovascular and metabolic dysfunctions, including hypertension, and that TES therapy is beneficial in reversing these conditions. Further understanding of the mechanisms by which TES and other androgens exert beneficial vs. detrimental effects on BP could lead to novel new therapeutic agents to treat HT in the aging male population and reverse symptoms associated with hypogonadism, such as hypertension and metabolic dysfunctions, which are important interactive risk factors for the development of CVD in men.

## Data Availability

Please contact the corresponding author for data requests.

## Abbreviations

ACE: Angiotensin-converting enzyme
Agt: Angiotensinogen
AR: Androgen receptor
AT_1_R: Angiotensin II type 1 receptor
BP: Blood pressure
BW: Body weight
CAD: Coronary artery disease
CVD: Cardiovascular disease
CsX: Castrated
DHT: Dihydrotestosterone
DRT: DHT-replacement therapy
HT: Hypertension
InT: Intact
LST: Losartan
RAS: Renin-angiotensin system
rt-PCR: Real-time polymerase chain reaction
SD: Sprague-Dawley
SV: Seminal vesicles
TES: Testosterone
Tfm: Testicular-feminized male
TRT: TES-replacement therapy
